# How is the minimal clinically important difference established in health-related quality of life instruments? Review of anchors and methods

**DOI:** 10.1186/s12955-020-01344-w

**Published:** 2020-05-12

**Authors:** Yosra Mouelhi, Elisabeth Jouve, Christel Castelli, Stéphanie Gentile

**Affiliations:** 1 Laboratoire de Santé Publique, Faculté de Médecine, Université Aix-Marseille, 3279 Marseille, EA France; 2 Service d’Evaluation Médicale, Assistance Publique - Hôpitaux de Marseille, Marseille, France; 3 Service Biostatistique Epidemiologie Santé Publique Innovation et Méthodologie (BESPIM), CHU Nîmes, Nîmes, France; 4 UPRES EA 2415 Aide à la décision médicale personnalisée, Faculté de Médecine, Université de Montpellier, Montpellier, France

**Keywords:** Health-related-quality of life, Minimal clinically important difference, Anchors-based methods, Distribution-based methods

## Abstract

**Background:**

The aim of this systematic review is to describe the different types of anchors and statistical methods used in estimating the Minimal Clinically Important Difference (MCID) for Health-Related Quality of Life (HRQoL) instruments.

**Methods:**

PubMed and Google scholar were searched for English and French language studies published from 2010 to 2018 using selected keywords. We included original articles (reviews, meta-analysis, commentaries and research letters were not considered) that described anchors and statistical methods used to estimate the MCID in HRQoL instruments.

**Results:**

Forty-seven papers satisfied the inclusion criteria. The MCID was estimated for 6 generic and 18 disease-specific instruments. Most studies in our review used anchor-based methods (*n* = 41), either alone or in combination with distribution-based methods. The most common applied anchors were non-clinical, from the viewpoint of patients. Different statistical methods for anchor-based methods were applied and the Change Difference (CD) was the most used one. Most distributional methods included 0.2 standard deviations (SD), 0.3 SD, 0.5 SD and 1 standard error of measurement (SEM). MCID values were very variable depending on methods applied, and also on clinical context of the study.

**Conclusion:**

Multiple anchors and methods were applied in the included studies, which lead to different estimations of MCID. Using several methods enables to assess the robustness of the results. This corresponds to a sensitivity analysis of the methods. Close collaboration between statisticians and clinicians is recommended to integrate an agreement regarding the appropriate method to determine MCID for a specific context.

## Introduction

Health-Related Quality of Life (HRQoL), a multidimensional construct that assesses several domains (e.g., physical, emotional, social), is an important Patient-Reported Outcome (PRO) in clinical trials as well as in routine clinical practice, and such information is also used by health policy makers in health care resource allocation and reimbursement decisions [1–3]. A PRO is defined as *“any report coming directly from patients about how they function or feel in relation to a health condition and its therapy”* [4]. Interpretation of changes in HRQoL scores of Patient-Reported Outcomes (PROs) is a challenge to the meaningful application of PRO measures in patient-centered care and policy [5].

Numerous clinical trials have established the importance of HRQoL in various diseases, and it is increasingly popular to evaluate generic and disease-specific HRQoL in clinical trials as a measure of patients’ subjective state of health [5, 6].

To be clinically useful, HRQoL instruments must demonstrate psychometric properties such as validity, reliability and responsiveness to change [7, 8]. Responsiveness to change is important for instruments designed to measure change over time. However, the statistical significance of a change in HRQoL scores does not necessarily imply that it is also clinically relevant [9–12]. Indeed, health policy makers need to present clinically meaningful results, to determine if the treatment is beneficial or harmful to their patients and also to know how to interpret and implement those results in their evidence-based method for clinical decision making [13]. Interpretation of clinical outcomes therefore should not be based solely on the presence or absence of statistically significant differences [14]. This highlights the need to define the minimal change in score considered relevant by patients and physicians, called ‘the Minimal Clinically Important Difference (MCID)’.

The MCID was first defined by Jaeschke [15] as ‘*the smallest difference in score in the domain of interest which patients perceive as beneficial and which would mandate, in the absence of troublesome side effects and excessive cost, a change in the patient’s management’*. MCID values are therefore important in interpreting the clinical relevance of observed changes, at both the individual and group levels. From the patient’s viewpoint, a meaningful change in HRQoL may be one that reflects a reduction in symptoms or improvement in function, however, a meaningful change for the physician may be one that indicates a change in the treatment or in the prognosis of the disease [16, 17].

Several methods have been developed, but no clear consensus exists regarding which methods are most suitable. An extensive review of available methods was published by Wells and colleagues and classified them into nine different methods [18].

Another review proposed three categories of methods for defining the MCID: distribution-based, opinion-based (relying upon experts) and anchor-based methods [19].

On one hand, anchor-based methods examine the relationship between a HRQoL measure with another measure of clinical change: the anchor [20]. Anchors can be derived from clinical outcomes (laboratory values, psychological measures, and clinical rating performance measures) or Patient-Reported Outcomes (PRO) (global health transition scale, patient’s self-reported evaluation of change) [20].

On the other hand, distribution-based methods use statistical properties of the distribution of outcome scores, particularly how the scores differ between patients. The distribution methods may use methods based on Standard Error of Measurement (SEM), Standard Deviation (SD), Effect Size (ES), Standardized Response Mean (SRM), Minimal Detectable Change (MDC), or Reliable Change Index (RCI) [20, 21].

The Delphi method has also been put forth in the literature. It involves the presentation of a questionnaire or interview to a panel of experts in a specific field for the purpose of obtaining a consensus [22]. The expert panel is provided with information on the results of a trial and are requested to provide their best estimate of the MCID. Their responses are averaged, and this summary is sent back with an invitation to revise their estimates. This process is continued until consensus is achieved [23].

To date, methods to determine MCID can be divided into two well-defined categories: distribution-based and anchor-based methods [20, 24–26]. These two methods are conceptually different. Distribution-based methods are the most used with a meaningful external anchor [20, 24–26]. Revicki et al. [21] recommended the usage of the anchor-based method to produce primary evidence for the MCID of any instrument and the distribution-based method to provide secondary or supportive evidence for that MCID.

The interest in estimation of MCID for HRQoL instruments has been increasing in recent years, and several reviews focused on estimates of MCID [20, 27–29]. MCID values have been shown to differ by population and study context as well as choice of anchors. This variability highlights the need to understand how the MCID was statistically established and what kind of anchors have been used, in order to facilitate its application in the Quality of Life field.

A systematic review was conducted to describe, from a structural literature search, the different types of anchors and statistical methods used in estimating the MCID for HRQoL instruments, either generic or disease-specific ones.

## Materials and methods

### Search strategy

A literature review was conducted in accordance with the preferred reporting items for systematic reviews and meta-analyses (PRISMA) [29].

To identify a large number of studies related to MCID, we performed a literature search on PubMed and Google scholar articles from 01 January 2010 to 31 December 2018 using the following request: (“*MCID” OR “MID” OR “minimal clinically important difference” OR “minimal important difference” OR “minimal clinically important change” OR “clinically important change” OR “minimal clinical important difference” OR “clinical important difference” OR “meaningful change”) AND (“health related quality of life”).*

A grey literature review was also performed.

We selected English and French language articles displaying an abstract and having included studies which (1) were original articles (i.e. reviews, meta-analysis, commentaries and research letters were not considered), (2) described anchors and statistical methods used to estimate the MCID in HRQoL instruments. We did not select the literature reviews, considered as secondary research articles, but we used the references of these reviews to search for other pertinent articles.

Two authors (YM and EJ) independently screened the study based on titles and abstracts. Then, authors (YM and EJ) obtained the selected full texts and read them to determine eligibility, and finally, the references in each of the retained articles were reviewed by YM and EJ for other relevant articles that might have been missed in the initial research.

### Data extraction and evaluation

For each included article, we collected data about:

- The year of publication,

- The study design. Four types were identified:

• *Prospective;*
*• Retrospective*;
*• Cross-sectional;*
*• Clinical trials.*
– The sample size (N);
– The disease;
– The HRQoL instrument: number of instruments used/subscale, generic and/or disease-specific;
– MCID estimation method: anchor and/or distribution, number of anchors, kind (subjective or clinical), cutoffs used, statistical methods, distribution criteria;
– The MCID value/range of each HRQoL instrument for each study.

The methodological quality of the included studies was independently assessed by two authors and disagreements were resolved by discussion. Articles that met eligibility criteria were grouped according to different clinical treatment areas. We then assessed MCID anchors and calculation methods, developed tables to display questionnaire names, calculation methods and type of anchors, MCID values by generic and disease-specific questionnaire.

## Results

### Selection process and general characteristics of included studies

**Fig. 1:**
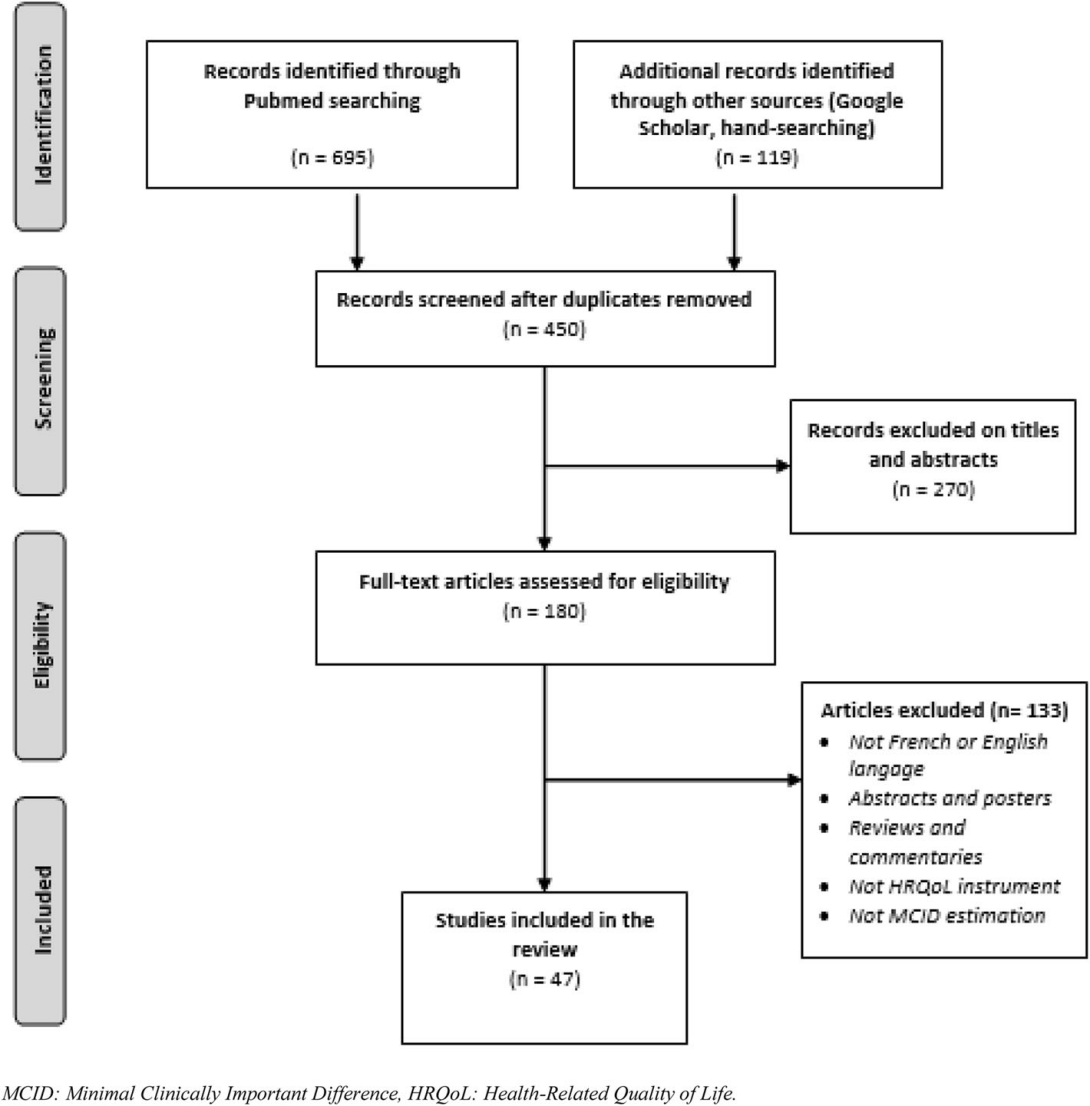
PRISMA Flow diagram of the literature search

The literature search identified 695 articles via PubMed, and 119 more articles were added with complementary research. After the selection process, this literature review included 47 articles (Fig. 1).

Our review provides an assessment of MCID for 6 generic and 18 disease-specific instruments (Table 1). Characteristics of the 47 included articles [30–76] are summarized in Table 2.

**Table 1:** HRQoL instruments: abbreviations and full names

Instrument-abbreviation	Instrument-full name
**Generic instruments**	
EQ-5D	European Quality of Life 5-Dimensions
SF-36	Short Form-36 Health Survey
SF-12	Short Form-12 Health Survey
SF-6D	Short-Form 6-Dimensions
WHOQOL- 100	World Health Organization Quality of Life assessment
15D	15-dimensions Quality of Life
**Disease-specific instruments**	
EORTC QLQ-C30	European Organization for Research and Treatment of Cancer Quality of Life- Questionnaire Core 30
EORTC QLQ-BM22	EORTC QLQ- bone metastases module
EORTC QLQ-BN20	EORTC QLQ- brain module
EORTC QLQ-C15-PAL	EORTC QLQ −15 palliative
EORTC GHS	EORTC Global Health Status (GHS)
UW-QOLQ	University of Washington Quality of Life Questionnaire
DLQI	Dermatology Life Quality Index
KQoL-26	Knee Quality of life 26-item
VascuQol	Vascular Quality Of Life
UCLA-PCI	UCLA prostate cancer index
SIS-16	Stroke Impact Scale
IWQOL-kids	Impact of Weight on Quality of Life-Kids
QLS /QOLS	Heinrichs–carpenter Quality of Life
PC-QOL	Parent Cough-Specific Quality of Life
PedsQL	Pediatric Quality Of Life Inventory
ASK Nasal-12	The Anterior Skull Base Nasal Inventory-12
PANQOL	The Penn Acoustic Neuroma QOL
PEmbQoL	Pulmonary Embolism Quality of Life

*HRQoL* health-related-quality of life

**Table 2:** General characteristics of the included studies (*N* = 47)

Reference (year)	Design	Patients, n	Age (mean or median)	Disease	HRQOL instrument					MCID estimation method	
					(n)	Instrument	Subscale/ dimension	Generic	Specific	Anchor-based	Distribution-based
***Oncology***											
Kvam AK et al. (2010) [30]	Prospective	239	66	Multiple myeloma	1	EORTC QLQ-C30	–	–	×	×	–
Kvam AK et al. (2011) [31]	Prospective	239	66	Multiple myeloma	3	EORTC QLQ-C30 EQ-5D 15D	–	×	×	×	×
Maringwa J et al. (2011) [32]	Randomized / controlled clinical trials	941	52	Brain cancer	2	QLQ-C30 QLQ-BN20	–	–	×	×	×
Maringwa J et al. (2011) [33]	Randomized / controlled clinical trials	812	57	Lung cancer	1	QLQ-C30	–	–	×	×	×
Zeng L et al. (2012) [34]	Prospective	93	63.4	Cancer with bone metastases	2	EORTC QLQ-C30 EORTC QLQ-BM22	–	–	×	×	×
Jayadevappa et al. (2012) [35]	Prospective	602	63.3	Prostate Cancer	2	SF-36 ULCA PCI	–	×	×	×	×
Den Oudsten BL et al. (2013) [36]	Prospective	223 = early stag 383 = benign breast problems	52.9 58.6	Early-stage breast cancer	1	WHOQOL- 100	–	×	–	×	×
Hong F et al. (2013) [37]	Prospective	Group1 = 191 Group 2 = 436	49 56	Any type of cancer	1	EORTC QLQ-C30	–	–	×	×	–
Bedard G et al. (2014) [38]	Prospective	369	57.7	Advanced cancer	1	EORTC QLQ-C30	–	–	×	×	×
Binenbaum Y et al. (2014) [39]	Prospective	1011	57	Oral cavity and Oropharynx Cancer	2	UW-QOLQ EORTC QLQ-C30	–	–	×	–	×
Sagberg LM et al. (2014) [40]	Prospective	164	56	Intracranial glioma surgery	1	EQ-5D	–	×	–	×	×
Wong E et al. (2015) [41]	Prospective	99	60.6	Advanced cancer with brain metastases	1	EORTC QLQ-BN20	–	–	×	×	×
Bedard G et al. (2016) [42]	Prospective	276	65.1	Advanced cancer	1	EORTC QLQ-C15-PAL	–	–	×	×	×
Yoshizawa K et al. (2016) [43]	Retrospective	710	66.7	Chronic NonCancer Pain	1	EQ-5D	–	×	–	×	–
Raman S et al. (2016) [44]	Randomized phase III trial	204	67.5	Bone metastases	2	EORTC QLQ-BM22 EORTC QLQ-C15-PAL	–	–	×	×	×
Quinten C et al. (2018) [45]	Prospective	741 = surgery 683 = chemotherapy	56.18 52.08	Cancer undergoing chemotherapy or surgery	1	EORTC Global Health Status (GHS)	–	–	×	×	×
Kerezoudis P et al. (2018) [46]	Prospective	1254	57.4	Patients with Vestibular Schwannoma	1	PANQOL	–	–	×	×	×
***Rheumatology/Musculoskeletal***											
Soer Rt al (2012) [47]	Prospective	151	51.9	Low back pain	1	EQ-5D	Categorical VAS	×	–	×	–
Parker SL et al. (2012) [48]	Retrospective	47	NR	Symptomatic pseudoarthrosis	2	SF-12 EQ-5D	SF-12 PCS	×	–	×	–
Parker SL et al. (2012) [49]	Retrospective	53	56.3	Revision surgery for same-level recurrent lumbar stenosis-associated back and leg pain	2	SF-12 EQ-5D	SF-12 PCS and MCS	×	–	×	–
Parker SL et al. (2013) [50]	Prospective	69	49.3	Anterior cervical discectomy and fusion	2	SF-12 EQ-5D	SF-12 PCS and MCS	×	–	×	–
Chuang LH et al. (2013) [51]	Clinical trial	Group1 = 121 Group 2 = 218	A = 45.5 GP = 48.1	Suspected internal derangement of the knee	1	KQoL-26	–	–	×	×	×
Díaz-Arribas MJ et al. (2017) [52]	Prospective	458	46.4	Low back pain	1	SF-12	PCS and MCS	×	–	×	–
Shi H et al. (2010) [53]	Prospective	67	70.2	Revision total hip arthoplasty	1	SF-36	–	×	–	–	×
Solberg T et al. (2013) [54]	Prospective	692	46	Lumbar disc herniation	1	EQ-5D	–	×	–	×	–
Carreon LY et al. (2013) [55]	Prospective	722 = primary procedures 333 = revision	60.8 50.9	Primary and revision lumbar fusion surgeries	1	SF-36	PCS	×	–	–	×
Asher AL et al. (2018) [56]	Prospective	441	62	Posterior lumbar surgery for grade I degenerative spondylolisthesis	1	EQ-5D	–	×	–	×	×
***Neurology/Neurovascular***											
Kwakkenbos et al. (2013) [57]	Prospective	211	NR	Systemic sclerosis	2	SF-6D EQ-5D	–	×	–	×	–
Kohn CG et al. (2014) [58]	Cross-sectional	3044	56.8	Multiple sclerosis	1	EQ-5D	–	×	–	–	×
Zhou F et al. (2015) [59]	Prospective	113	57.6	Cervical spondylotic myelopathy	1	SF-36	PCS and MCS	×		×	–
Fulk GD et al. (2010) [60]	Prospective	36	60.9	Stroke	1	SIS-16	–	–	×	×	–
Frans FA (2014) [61]	Prospective	127	67	Critical Limb Ischemia	1	VascuQol	–	–	×	×	×
Kim SK et al. (2015) [62]	Prospective	487	68.3	Stroke	2	SF-6D EQ-5D	–	×	–	×	–
Chen P et al. (2016) [63]	Prospective	65	52.8	Stroke	1	EQ-5D	EQ-5D-5 L	×	–	×	×
Yuksel S et al. [2018] [64]	Prospective	185 = surgical patients 86 = nonsurgical patients	52.4 44.9	spinal deformity	1	SF-36	PCS and MCS	×	–	×	×
***Psychiatry/Gastroenterology/Dermatology***											
Le QA et al. (2013) [65]	Randomized, controlled trial	200	37.5	Post-traumatic stress disorder	1	EQ-5D	–	×	–	×	×
Thwin SS et al. (2013) [66]	Randomized clinical trials	350	50.7	Schizophrenia	1	QOLS	–	–	×	×	–
Falissard B et al. (2015) [67]	Randomized	351	38.6	Schizophrenia	1	QLS	–	–	×	×	–
Stark RG et al. (2010) [68]	Cross-sectional	502	42	Bowel inflammatory disease	1	EQ-5D	–	×	–	×	–
Basra MK et al. (2015) [69]	Prospective	192	38.7	Inflammatory skin disease	1	DLQI	–	–	×	×	–
***Pediatry***											
Modi AC et al. (2011) [70]	Cross-sectional	263	15.1	Obesity/weight	1	IWQOL-kids	–	–	×	–	×
Newcombe PA et al. (2011) [71]	Prospective	34	26.5 months	Pediatric Chronic Cough	1	PC-QOL	–	–	×	×	×
Hilliard ME et al. (2013) [72]	Prospective	5004	12.5	Diabetes type 1 and 2	1	PedsQL	–	–	×	–	×
***Rhinology***											
Gravbrot N et al. (2018) [73]	Prospective	–	–	Patients undergoing transsphenoidal surgery	1	ASK Nasal-12	–	–	×	×	×
Hoehle LP et al. (2018) [74]	Prospective	203	54.1	Chronic rhinosinusitis (CRS)	1	EQ-5D	HUV VAS	×	–	×	×
***Pulmonary***											
Akaberi A et al. (2018) [75]	Prospective	82	49.4	Pulmonary embolism	1	PEmbQoL	–	–	×	×	×
***More than one disease***											
Alanne S et al. (2015) [76]	Prospective	4903	60	16 diseases	1	15D	–	×	–	×	–

-: Not used; × used, *MCID* minimal clinically important difference

More than half of the studies were prospective (*n* = 34, 72.3%), 3 retrospective (6.4%), 3 cross-sectional (6.4%) and 7 were clinical trials (14.9%). Nearly 40% of studies have been conducted in the field of oncology.

In addition, 75% of studies estimated the MCID for only one HRQoL instrument while 25% for two or three instruments. Twenty-two (46.8%) studies focused only on a generic HRQoL instrument, 23 (48.9%) studies only on a disease-specific HRQoL instrument, and 2 (4.3%) studies combined both.

### Methods of MCID estimation

In this review, 18 (38.3%) of the included studies used only anchor-based methods to estimate the MCID; 6 (12.8%) studies used only distribution-based methods, and 23 (48.9%) combined both to provide more accurate estimates (Table 2).

### Anchor-based methods

#### Type of anchors

Among the 41 studies using anchor-based methods, 36 studies applied non-clinical anchors and only 5 studies applied clinical ones. Anchors adopted in the included studies are presented in Table 3. For each of these anchors, authors predefined different cutoffs that vary depending on the study context.

**Table 3:** MCID methods estimation: anchors and statistical methods

Reference	Anchor-based					Distribution-based	
	n_**1**_	Anchor (s)	Viewpoint	Cutoffs used	Statistical methods	n_**2**_	Distribution criteria
Kvam AK et al. [30]	1	Global Rating of Change (GRC: 1–7)	Patient	Improvement: ‘much better, moderately better and a little better’ Deterioration: ‘a little worse, moderately worse and much worse’	CD	–	–
Kvam AK et al. [31]	1	Global Rating of Change (GRC: 1–7)	Patient	Improved: ‘much better, moderately better and a little better’ Deteriorated: ‘a little worse, moderately worse and much worse’	AC	2	0.2 SD, 0.5 SD
Maringwa J et al. [32]	2	World Health Organization performance status (WHO PS: 0–4) Mini-mental state examination (MMSE: 1–30)	Clinical	WHO PS: ± 1 MMSE: + 4 or + 5	CD	4	0.2SD, 0.3SD, 0.5SD, SEM
Maringwa J et al. [33]	2	World Health Organization performance status (WHO PS:0–4) Weight change	Clinical	WHO PS: ± 1 Weight gain: < 20%	CD	3	0.2SD, 0.5SD, SEM
Zeng L et al. [34]	1	Karnofsky Performance Status (KPS: 0–100)	Clinical	± 10	CD	4	0.2 SD, 0.3 SD, 0.5 SD, SEM
Jayadevappa et al. [35]	2	Health Transition Item of the SF-36 (HTI: NR) The patient-reported physical signs/symptoms (NR)	Patient	‘General health’ ‘More tired’	Linear regression	3	1SEM, 0.3SD, 0.5SD
Den Oudsten BL et al. [36]	1	General Health and Overall QoL (− 9 to + 9)	Patient	‘Small positive change’: 2 ≤ C ≤ 3 ‘Small negative change’: − 3 ≤ C ≤ -2	CD	2	1SEM, 0.5SD
Hong F et al. [37]	1	The Subject Significance Questionnaire (SSQ: − 3 to + 3)	Patient	NR	Linear regression	–	–
Bedard G et al. [38]	2	Overall health (1–7) Overall QoL (1–7)	Patient	Overall health: + 2 Overall QoL: + 2	CD	4	SEM, 0.2SD, 0.3SD, 0.5SD
Binenbaum Y et al. [39]	–	–	–	–	–	1	0.5SD
Sagberg LM et al. [40]	1	Karnofsky Performance Status (KPS: 0–100)	Clinical	± 10	AC	1	0.5SD
Wong E et al. [41]	1	Overall QoL (1–7)	Patient	Overall QoL: 1	CD	4	SEM, 0.2SD, 0.3SD, 0.5SD
Bedard G et al. [42]	1	Overall QoL (1–7)	Patient	Overall QoL: + 2	CD	4	SEM, 0.2SD, 0.3SD, 0.5SD
Yoshizawa K et al. [43]	1	Physician’s global impression of treatment effectiveness (PGI: NR)	Physician	‘Effective’ vs ‘not effective’	ROC	–	–
Raman S et al. [44]	1	Overall QoL (1–7)	Patient	+ 10	CD	4	0.2SD, 0.3SD, 0.5SD, SEM
Quinten C et al. [45]	3	The 15-item Geriatric Depression Scale (GDS15) (0 to 4) Visual Analogue Scale (VAS) for Fatigue (0 to 10) ECOG Performance Status (PS) (0 to 4)	Clinical	Improvement: ‘improved’ vs ‘stabe’ Deterioration: ‘no ‘stabe’ vs ‘worse’	CD	1	0.2SD
Kerezoudis P et al. [46]	1	Health Transition Item (1–5)	Patient	‘Somewhat better’ or ‘Somewhat worse	CD	2	0.5SD, 1SEM
Soer Rt al [47]	2	Pain Disability Index (PDI: 1–10) Global perceived effect (GPE: 1–7)	Patient	PDI: -9 GPE: +4	ROC	–	–
Parker SL et al. [48]	2	Health Transition Item (HTI: 1–4) Patient’s satisfaction after the surgery	Patient	HTI: ‘Slightly better’ or Markedly better’ Patient’s satisfaction: ‘Yes’	ROC, AC, MDC, CD	–	–
Parker SL et al. [49]	2	Health Transition Item of SF-36 (HTI: 1–4) Patient’s satisfaction after the surgery	Patient	HTI: ‘Slightly better’ or ‘Markedly better’ Patient’s satisfaction: ‘Yes’	ROC, AC, MDC, CD	–	–
Parker SL et al. [50]	1	North America Spine Society (NASS) patient Satisfaction Scale (1–4)	Patient	‘The treatment met my expectations’	ROC, AC, MDC, CD	–	–
Chuang LH et al. [51]	1	Health Transition Item of the SF-36 (HTI: 0–15)	Patient	‘A little better’ or ‘Somewhat better’	ROC	2	1SEM, MDC
Díaz-Arribas MJ et al. [52]	1	Self-reported health status change between baseline and 12 month-assessment (NR)	Patient	‘Completely recovered’ or ‘improved’	ROC, AC, MDC, CD	–	–
Shi H et al. [53]	–	–	–	–	–	1	0.5SD
Solberg T et al. [54]	1	Global Perceived Scale Of Change (1–7)	Patient	‘Completely recovered’ or ‘much improved’	ROC	–	–
Carreon LY et al. [55]	–	–	–	–	–	1	MDC
Asher AL et al. [56]	1	North America Spine Society (NASS) society Satisfaction Scale (1–4)	Patient	‘Satisfied’ and ‘not satisfied’ groups	AC	3	0.5SD,1SEM,MDC
Kwakkenbos et al. [57]	2	Global Rating of Change (GRC) (1–7) The Health Assessment Questionnaire-Disability Index (HAQ-DI:0–3)	Patient	GRC = 2 ‘somewhat better’ or 4 ‘somewhat worse’ MCID of HAQ-DI: + 0.22	CD	–	–
Kohn CG et al. [58]	–	–	–	–	–	3	1SEM, 0.5SD, 0.33SD
Zhou F et al. [59]	1	Health Transition Item of the SF-36 (HTI: 1–4)	Patient	‘Slightly better’ or Markedly better’	ROC, AC, MDC, CD	–	–
Fulk GD et al. [60]	2	Global Rating of Change (GRC: − 7 to 7) scores	Patient + Physician	+ 5	ROC	–	–
Frans FA [61]	1	The change in Fontaine classification (1–4)	Physician	Improvement: ‘improved’ vs ‘no change’ Deterioration: ‘worse’ vs ‘no change’	AC	1	0.5SD
Kim SK et al. [62]	2	The modified Rankin scale (MRS: 0–5) The Barthel index (BI: 0–20)	Patient	Improvement: ‘Minimally better’ Deterioration: ‘Minimally worse’	CD	–	–
Chen P et al. [63]	1	The perceived recovery score of the Stroke Impact Scale 3.0 (NR)	Patient	10–15%	CD	1	0.5SD
Yuksel S et al. [64]	1	Global Rating of Change (GRC: −7 to 7) scores	Patient	Patients perceiving an improvement as opposed to those who do not (i.e. worse or unchanged)	Latent class analysis (LCA)	1	MDC
Le QA et al. [65]	2	Clinical Global Impression Improvement (CGI:1–7) The symptom Scale-Interview (PSS-I)	Physician	CGI: 3 or less PSS-I: 23 or less	ROC Regression analysis	2	0.2SD, 0.5SD
Thwin SS et al. [66]	1	Clinical Global Impressions Improvement (CGI-I: 1–7)	Physician	CGI-I: 1	Equipercentile method	–	–
Falissard B et al. [67]	1	Clinical Global Impressions of Severity (CGI-S: 1–7)	Physician	‘Slightly improved’	CD	–	–
Stark RG et al. [68]	1	Patient’s perceived improvement after the treatment (NR)	Patient	Improvement: ‘better’ Deterioration: ‘worse’	Regression analysis	–	–
Basra MK et al. [69]	1	Global Rating of Change (GRC: − 7 to + 7)	Patient	Small change ±2, ±3	CD	–	–
Modi AC et al. [70]	–	–	–	–	–	1	SEM
Newcombe PA et al. [71]	1	Verbal category descriptive score (VCD: 0–5)	Patient	+ 1	CD	3	ES, SEM, 0.5SD
Hilliard ME et al. [72]	–	–	–	–	–	1	1SEM
Gravbrot N et al. [73]	2	The 2-wk postoperative overall nasal functioning item The 2-wk postoperative Short Form Health Survey 8 bodily pain item	Patient	1 unit	CD	2	ES, 0.5SD
Hoehle LP et al. [74]	1	A question related to change in general health-related QOL (1–5)	Patient	‘About the same’ compared to ‘A little better’	CD ROC	1	0.5 SD
Akaberi A et al. [75]	2	General QoL using SF-36 PCS and MCS (0–100) Dyspnea severity (0–120)	Patient	General QoL = a t least a 4-point Change Dyspnea severity = t least a 5-point change	Repeated-measures mixed-effect models	1	ES
Alanne S et al. [76]	1	Subjective five-category global assessment scale (GAS: 1–5)	Patient	Improvement: ‘Slightly better’ Deterioration: ‘Slightly worse	ROC	–	–

-: Not used, *n*_*1*_ number of anchors, *n*_*2*_ number of distribution criteria, *MCID* minimal clinically important difference, *QoL* quality of life, *AC* average change, *MDC* minimal detectable change, *CD* change difference, *ROC* receiver operating curve, *ES* effect size, *SD* standard deviation, *SEM* standard error of measurement

Among the 36 studies using non-clinical anchors, 30 of them chose anchors from the viewpoint of patients, 5 from the viewpoint of physicians and 1 from the viewpoint of both.

Anchors from patient point of view are based on questions to assess how a patient feels about his or her current health status over time or on Patient-Reported Outcomes (PRO):

- The Global Rating of Change (GRC) scale (*n* = 6): used by authors on a 15-point ordinal scale or on a 7-point scale.
- Global and transition questions: the most common was the Health Transition Item (HTI) of the SF-36 (n = 6). The other questions related to the instruments were differently applied and are described in detail in Table 3.
- PRO such as Pain Disability Index, the perceived recovery score of the Stroke Impact scale, the Symptom Scale-Interview …
- Other scales such as the Modified Rankin Scale, the Barthel Index …

Five studies used a physician point of view anchor:

- The dichotomous physician’s global impression of treatment effectiveness (PGI): this question was a discrete choice of “effective” or “not effective” treatment.
- The Clinical Global Impressions scales: Improvement (CGI-I) or severity (CGI-S).
- The change in Fontaine classification: rated on a 4-point scale (much improved, improved, unchanged and worse).

Four studies used a Performance Status (PS) as clinical anchor:

- The Karnofsky Performance Scale (KPS).
- The World Health Organization Performance Status (WHO PS), combined with Mini-Mental State Exam (MMSE) or Weight change.

#### Statistical methods used for anchors-based methods

**Fig. 2:**
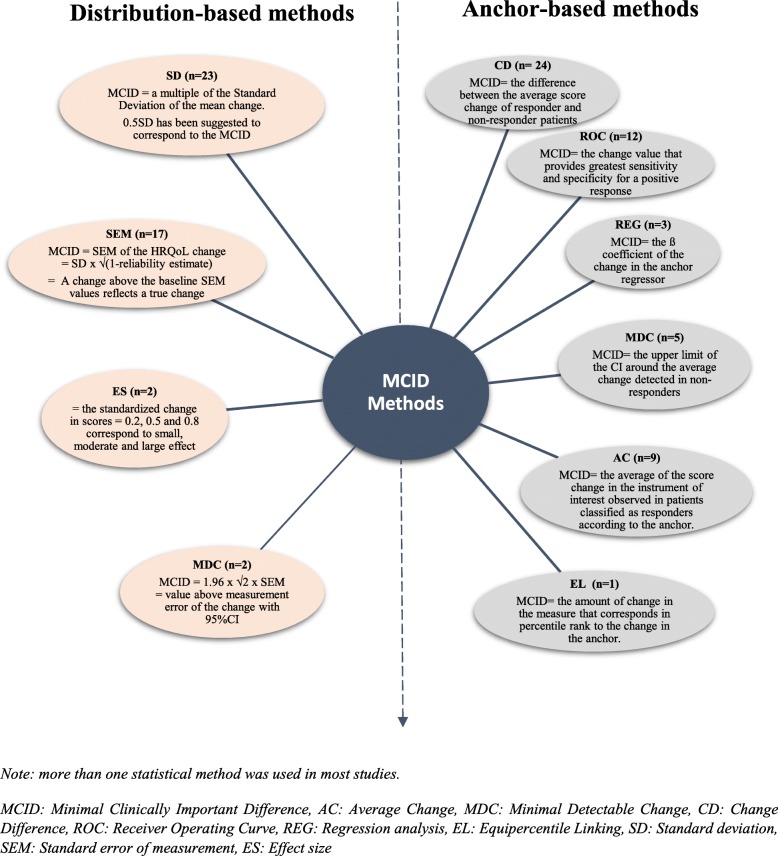
Review of Statistical methods applied in the included studies

Among the 41 studies using anchor-based methods, 36 applied only one statistical method. These methods were Change Difference (CD), Receiver Operating Curve (ROC), Regression analysis (REG), Average Change (AC) and Equipercentile Linking (EL). Furthermore, 5 studies combined many of these methods (Fig. 2, Table 3).

Mostly, determination of MCID was based on the calculation of a change of HRQoL score between two times, from a baseline (longitudinal study).

Among the 36 studies using only one statistical method, the most common were (Fig. 2):

- The CD: MCID was identified by the difference between the average of HRQoL score change of responder patients (defined by the anchor) and the average score change of non-responder patients.
- The ROC: created by plotting the sensitivity of the instrument (the true positive rate) against the specificity (the false positive rate). Some studies [43, 51, 54, 60, 76] identified the MCID as the upper corner of the curve, and other studies [45, 65] identified the MCID as the point of the receiver operating characteristics curve in which sensitivity and specificity are maximized (Maximum (Sensibility+Specificity-1), Youden index). The area under the curve (AUC) was always calculated to measure the instrument responsiveness, suggesting AUC values upper than 0.7.
- The regression analysis of HRQoL score (or change) by anchor as regressor: authors defined the MCID as the coefficient estimate of the anchor.
- The method of AC: by relating the average of the HRQoL score change observed in patients classified as responders according to the anchor.
- The EL: the value of change in the HRQoL score that corresponds in percentile rank to the change in the anchor is interpreted as the MCID.

All 5 studies combining 4 methods: CD, ROC, AC and MDC methods.

- The MDC (Minimal Detectable Change) is defined as the upper limit of the 95% confidence interval (CI) of the average change detected in non-responders.

Two of these 5 studies [49, 50] chose the MDC as the most appropriate method to identify the MCID, since it was the only method to provide a threshold above the 95% Confidence Interval of the unimproved cohort (greater than the measurement error). The three other studies [46, 52, 59] did not find a difference between the 4 methods to determine the true value of MCID.

### Distribution-based methods

Among the 29 studies using distribution-based methods, 13 applied only one method, while most studies (*n* = 16) combined more than one distributional method (Table 3).

The most common were (Fig. 2):

- Multiples of Standard Deviation were used as MCID: 0.5SD, 0.3SD, 1/3SD, 0.2SD. Most authors (*n* = 22) used 0.5 Standard Deviation of the HRQoL mean change score between two time points. Frequently 2 or 3 multiples of 0.5SD, 0.3SD and/or 0.2SD (*n* = 12) were used, only one with 1/3SD. Multiples of SD were related to effect size: 0.2SD (small effect) to 0.5SD (median effect).
- The Standard Error of Measurement (SEM): calculated by the formula SEM = SD √(1-r) where r is a reliability estimation of HRQoL score (ratio of the true score variance to the observed score variance or internal consistency measure as Cronbach’s alpha). This characteristic of precision was frequently used (*n* = 16), and associated with multiple SD.
- The Effect Size (ES): used in one study and represents the standardized HRQol score change. Common statistic is calculated by the ratio of the score change divided by the standard deviation of the score.
- The Minimal Detectable Change (MDC): used in two studies and calculated as 1.96 × √2 × SEM (for a 95% confidence interval). The MDC represents the smallest change above the measurement error with a confidence interval.

Thereby, most studies (*n* = 16) combined the fractionations including 0.2 SD, 0.3 SD or 0.5 SD and/or 1 SEM in order to provide a range of MCID values (Table 3).

### MCID values

As shown in the supplementary file (see Additional file 1), variability in MCID results were observed for each HRQoL instrument, depending on:

- Pathology: MCID for SF-36 PCS ranged from 4.9 to 5.21 [55] and from 4.09 to 9.62 [59] for Rheumatology and neurology population, respectively.
- Methodology: even for the same pathology, MCID values were variable. For example, for EQ-5D, MCID values, using anchor and/or distribution-based methods, varied from 0.01 to 0.39 for patients with rheumatology/musculoskeletal disorders [47–50, 54] and from 0.08 to 0.15 for oncology patients [31, 40, 43]. For patients with psychology disorders, MCID ranged from 0.05 to 0.08 using anchor-based method, and 0.04 to 0.1 using distribution-based method [65].
- Statistical method: MCID for EORTC QLQ-C30 in oncology patients ranged from − 27 to 17.5 using CD method [30], − 12 to 8 using AC method [31] and − 11.8 to 11.8 using regression analysis [37].
- Change direction: some studies calculated MCID irrespective of the change direction or separately for improvement and deterioration without major impact on MCID values and did not find a major impact on MCID values. The WHOQOL-100, for example, was assessed in early-stage breast cancer population [36], MCID for improvement ranged from 0.51 to 1.27 and for decline from − 1.56 to − 0.71.

## Discussion

Our systematic review identified 47 studies reporting anchors and statistical methods to estimate MCID for generic and disease-specific HRQoL instruments. This review pointed out that the interest of MCID in HRQoL instruments has been increasing in the recent years and the largest work has been done in the field of oncology disorders.

Most studies used anchor-based methods in our review (*n* = 41), either alone or in combination with distribution-based methods. As discussed by Gatchel and Mayer [77], anchor-based methods are good depending on the choice of the external criteria as well as the methodology used.

We observed multiple anchors chosen by authors, and the most common anchors were non-clinical and from the viewpoint of patients in order to assess how a patient feels about his or her current health status over time. These anchors are well-studied and applicable to a wide range of patients [78]. However, patients may be aware that the phase of their disease is deteriorating, thus they will conclude that their HRQoL is similarly deteriorating. Furthermore, the patients’ subjective experiences are related to the way in which people construct their memories. It is hard for people to accurately recall a previous health state; they will rather create an impression of how much they have changed by considering their present state and then retrospectively applying some idea of their change over time. Hermann [79] described the problem of “recall bias” where events intervening between the anchor points influence the recall of the original status, while Schwartz and Sprangers [80] described “response-shift” where a patient’s response is influenced by a changing perception of their context.

Clinical anchors were not widely applied in the included studies. Changes in Performance Status (PS), in particular the KPS and the WHO PS, were chosen by authors because of accessibility and interpretability [25]. As they do not provide MCID values per se [81], clinical anchors were applied in our review with distribution criterion. However, we did not find any combination with another subjective anchor.

Authors recommended the usage of multiple independent anchors [20]. Anchors must be easily interpretable, widely used and at least moderately correlated with the instrument being explored [8, 32, 33]. According to Cohen’s [82], 0.371 was recommended as a correlation threshold to define an important association. However, anchor-based methods may be vulnerable to recall bias, and as was evident in our review, different anchors may produce widely different estimates for the same HRQoL instrument.

Cut-offs for different anchors were differently assigned by authors. Even for the same anchor, many cutoffs were used. There is no agreement on the exact cutoffs for anchors, they are generally assigned for the purposes of research and depending on study context and anchor used [29].

Once the anchor has been chosen, different statistical methods were applied to estimate the MCID. The most established method in our review was the mean change score, also called the Change Difference (CD) method. This latter is defined as the mean change of patients who improved and, therefore, authors can set its cutoffs on the basis of the change score of patients who were shown to have had a small, moderate, or large change. MCID corresponds to the difference between two adjacent levels on the anchor. MCID would depend on the number of levels on the anchor: the larger the number of levels, the smaller the difference between two adjacent levels, and the smaller the MCID [83].

Each of the statistical methods has its specific concepts and produces a MCID value different from the other methods. Some authors pointed out that the largest threshold value is most often generated from the average change method, whereas the smallest threshold from the CD and MDC methods [48–50].

Therefore, the usage of patient point of view’ anchors by most studies may be explained by the lack of satisfying objective scales, which incite the usage of subjective anchors in first place. In addition, perhaps the CD method is simple to apply by many authors, but we cannot affirm that this is the most relevant method. We conclude that there are many faces to the MCID, it is not a simple concept, nor simple to estimate.

In addition, distribution-based methods, derived from statistical analysis, were also applied in few studies. In accordance with literature [24–29], most often fractionations in our review include 0.2 SD, 0.3 SD, 0.5 SD and 1 standard error of measurement (SEM).

Some studies determined that MCID corresponded closest to the 0.5 SD estimate. The 0.5 SD was the value in which most meaningful changes fall, as previously proven in a study by Norman et al. [84].

Distribution-based methods also produced different values of MCID depending on the distributional criterion. Nevertheless, distribution-based methods do not address the question of clinical importance and ignore the aim of MCID, which is to define the clinical importance distinctly from statistical significance. Authors recommended the usage of these methods when anchor-based calculations are unavailable [20].

In this review, MCID values were defined for Patient-Reported Outcomes (PRO) measuring HRQoL using the two methods: anchor and distribution-based methods. Some studies had been developed to determine MCID for the Patient-Reported Outcomes Measurement Information System (PROMIS) instruments, using the same reported statistical methods. In recent years, the PROMIS Network (www.nihpromis.org), a National Institutes of Health Roadmap Initiative, has advanced PRO measurement by developing item banks for measuring major self-reported health domains affected by chronic illness. Therefore, further studies should be developed to determine the meaningful change in HRQoL for PROMIS.

Summing up, we did not observe a single MCID value for any HRQoL instrument in our review. Several factors may influence this variability. On one hand, we found many available methods that produced many MCID values for the same HRQoL instrument. Authors applied, for the same instrument and in the same cohort, four different methods and reported four different MCID values [48–50, 52, 59], which suggest that variation could be explained further than differences in disease severity or disease group since the same cohort of patients was analyzed. On the other hand, even with the same methodology for the same instrument, MCID values vary since the variability may be related to study population, in particular, patient demographics and patient baseline status. Wang et al. [85] stated that MCID scores are context-specific, depending on patient baseline and demographic characteristics. Therefore, factors affecting MCID values are specific to the population being studied and are non-transferable across patient groups, also related to the multiple reported conceptual and methodological differences.

Our review exhibits some limitations that deserve mention. First, the search strategy only focused on Pubmed and Google scholar, which might have caused the loss of some papers. However, the inclusion of grey literature is a useful source of relevant information, which ensures a certain standard of quality of the selected papers. In addition, our review was limited to the nine last years, which can also lead to the loss of some papers. To our knowledge, there is no review published recently to define MCID in the QoL field, our objective was therefore to provide researchers the new statistical methods to be applied for further researches.

The following question remains to be answered “which is the best method for MCID”? Sloan J (2005) [86] stated that *“there are many methods available to ascertaining an MCID, none are perfect, but all are useful”.* The MCID can be best estimated using a combination of anchor and distribution measures to triangulate toward a single value. Using several methods enables to assess the robustness of the results. This corresponds to a sensitivity analysis not on the data but on the methods. Anchor-based methods should be used as primary measures with distribution methods as supportive measure.

## Conclusion

We conclude that many methods have become available, which lead to different estimations of MCID. MCID should be based on the context of each clinical study. Therefore, in order to stay cautious while interpreting MCID in the field of Quality of Life, close collaboration between statisticians and clinicians may be critical and necessary in order to integrate an agreement regarding the appropriate method to determine MCID. Moreover, as performed for the data, a sensitivity analysis on method, ie performing the analysis with several methods is highly recommended to assess the robustness of the results.

## Supplementary information

**Additional file 1.**

## Data Availability

Not applicable. Data sharing not applicable to this article as no datasets were generated or analysed during the current study.

## Abbreviations

AC: Average change
AUC: Area under the curve
CD: Change difference
CGI-I: Clinical global impressions improvement
CGI-S: Clinical global impressions severity
EL: Equipercentile linking
ES: Effect size
GRC: Global rating of change
HRQoL: Health-related quality of life
HTI: Health transition item
KPS: Karnofsky performance scale
QoL: Quality of life
MCID: Minimal clinically important difference
MCS: Mental component summary
MDC: Minimal detectable change
MMSE: Mini-mental state exam
PCS: Physical component summary
PRO: Patient-reported outcome
PRISMA: Preferred Reporting items for systematic reviews and meta-analyses
PROMIS: Patient-reported outcomes measurement information system
PS: Performance status
REG: Regression analysis
ROC: Receiver operating curve
SD: Standard deviations
SEM: Standard error of measurement
SF-36: The Short-Form 36
WHO PS: World Health Organization Performance Status

## References

[1] Patrick DL, Chiang YP (2000). Measurement of health outcomes in treatment effectiveness evaluations: conceptual and methodological challenges. Med Care.

[2] Revicki DA, Osoba D, Fairclough D (2000). Recommendations on health-related quality of life research to support labeling and promotional claims in the United States. Qual Life Res.

[3] Testa MA, Simonson DC (1996). Assessment of quality-of-life outcomes. N Engl J Med.

[4] Lipscomb J, Gotay CC (2007). Patient-reported outcomes in cancer: a review of recent research and policy initiatives. CA Cancer J Clin.

[5] Schipper H, Clinch J, Powell V, Spilker B (1990). Definitions and conceptual issues. Quality of life assessments in clinical trials.

[6] Fiebiger W, Mitterbauer C, Oberbauer R (2004). Health-related quality of life outcomes after kidney transplantation. Health Qual Life Outcomes.

[7] Guyatt G, Walter S, Norman G (1987). Measuring change over time: assessing the usefulness of evaluative instruments. J Chronic Dis.

[8] Guyatt GH, Osoba D, Wu AW, Wyrwich K (2002). Methods to explain the clinical significance of health status measures. Mayo Clin Proc.

[9] Wright JG (1996). The minimal important difference: who’s to say what is important. J Clin Epidemiol.

[10] Wright A, Hannon J (2012). Clinimetrics corner: a closer look at the minimal clinically important difference (MCID). J Manual Manipulative Ther.

[11] Batterham AM, Hopkins WG (2006). Making meaningful inferences about magnitudes. Int J Sports Physiol Perform.

[12] Page P (2014). Beyond statistical significance: clinical interpretation of rehabilitation research literature. Int J Sports Phys Ther.

[13] Kristensen N, Nymann C (2016). Implementing research results in clinical practice- the experiences of healthcare professionals. BMC Health Serv Res.

[14] Juniper EF, Guyatt GH (1994). Determining a minimal important change in a disease-specific quality of life questionnaire. J Clin Epidemiol.

[15] Jaeshke R, Singer J, Guyatt G (1989). Measurement of health status. Ascertaining the minimal clinically important difference. Control Clin Trials.

[16] Cook CE (2008). Clinimetrics corner: the minimal clinically important change score (MCID): a necessary pretense. J Man Manipulative Ther..

[17] Crosby RD, Kolotkin RL, Williams GR (2003). Defining clinically meaningful change in health-related quality of life. J Clin Epidemiol.

[18] Wells G, Beaton D, Shea B (2001). Minimal clinically important differences: review of methods. J Rheumatol.

[19] Lassere MN, van der Heijde D, Johnson KR (2001). Foundations of the minimal clinically important difference for imaging. J Rheumatol.

[20] Rai SK, Wazdany J (2015). Approaches for estimating minimal clinically important difference in systemic lupus erythematosus. Arthritis Res Ther.

[21] Revicki D, Hays RD, Cella D, Sloan J (2008). Recommended methods for determining responsiveness and minimally important differences for patient-reported outcomes. J Clin Epidemiol.

[22] Black N, Murphy M, Lamping D, McKee M, Sanderson C, Askham J (1999). Consensus development methods: a review of best practice in creating clinical guidelines. J Health Serv Res Policy.

[23] McKenna HP (1994). The Delphi technique: a worthwhile research approach for nursing?. J Adv Nurs.

[24] Norman GR, Sridhar FG, Guyatt GH, Walter SD (2001). Relation of distribution-and anchor-based approaches in interpretation of changes in health-related quality of life. Med Care.

[25] Copay AG, Subach BR (2007). Understanding the minimum clinically important difference: a review of concepts and methods. Spine J.

[26] Wyrwich KW, Bullinger M, Aaronson N (2005). Estimating clinically significant differences in quality of life outcomes. Qual Life Res.

[27] Keurentjes JC, Van Tol FR (2012). Minimal clinically important differences in health-related quality of life after total hip or knee replacement: a systematic review. Bone Joint Res.

[28] Coretti S, Ruggeri M, McNamee P (2014). The minimum clinically important difference for EQ-5D index: a critical review. Expert Rev Pharmacoeconomics Outcomes Res.

[29] Jayadevappa R, Cook R, Chhatre S (2017). Important difference to infer changes in health related quality of life-a systematic review. J Clin Epidemiol.

[30] Kvam AK, Wisloff F (2010). Minimal important differences and response shift in health-related quality of life; a longitudinal study in patients with multiple myeloma. Health Qual Life Outcomes.

[31] Kvam AK, Fayers PM (2011). Responsiveness and minimal important score differences in quality-of-life questionnaires: a comparison of the EORTC QLQ-C30 cancer specific questionnaire to the generic utility questionnaires EQ-5D and 15D in patients with multiple myeloma. Eur J Haematol.

[32] Maringwa J, Quinten C, King M, Ringash J (2011). Minimal clinically meaningful differences for the EORTC QLQ-C30 and EORTC QLQ-BN20 scales in brain cancer patients. Ann Oncol.

[33] Maringwa JT, Quinten C, King M (2011). Minimal important differences for interpreting health-related quality of life scores from the EORTC QLQ-C30 in lung cancer patients participating in randomized controlled trials. Support Care Cancer.

[34] Zeng L, Chow E, Zhang L (2012). An international prospective study establishing minimal clinically important differences in the EORTC QLQ-BM22 and QLQ-C30 in cancer patients with bone metastases. Support Care Cancer.

[35] Jayadevappa R (2012). Comparison of distribution- and anchor-based approaches to infer changes in health-related quality of life of prostate Cancer survivors. Health Serv Res.

[36] Den Oudsten BL, Zijlstra WP (2013). The minimal clinical important difference in the World Health Organization quality of life Instrument-100. Support Care Cancer.

[37] Hong F, Bosco JLF, Bush N, Berry DL (2013). Patient self-appraisal of change and minimal clinically important difference on the European organization for the research and treatment of cancer quality of life questionnaire core 30 before and during cancer therapy. BMC Cancer.

[38] Bedard G (2014). Minimal important differences in the EORTC QLQ-C30 in patients with advanced Cancer. Asia-Pac J Clin Oncol.

[39] Binenbaum Y, Amit M (2014). Minimal clinically important differences in quality of life scores of oral cavity and oropharynx cancer patients. Ann Surg Oncol.

[40] Sagberg LM, Jakola AS (2014). Quality of life assessed with EQ-5D in patients undergoing glioma surgery: what is the responsiveness and minimal clinically important difference?. Qual Life Res.

[41] Wong E, Zhang L, Kerba M (2015). Minimal clinically important differences in the EORTC QLQ-BN20 in patients with brain metastases. Support Care Cancer.

[42] Bedard G, Zeng L, Zhang L (2016). Minimal important differences in the EORTC QLQ-C15-PAL to determine meaningful change in palliative advanced cancer patients. Asia Pac J Clin Oncol.

[43] Yoshizawa K, Kobayashi H, Fujie M (2016). Estimation of minimal clinically important change of the Japanese version of EQ-5D in patients with chronic noncancer pain: a retrospective research using real-world data. Health Qual Life Outcomes.

[44] Raman S, Ding K, Chow E (2016). Minimal clinically important differences in the EORTC QLQ-BM22 and EORTC QLQ-C15-PAL modules in patients with bone metastases undergoing palliative radiotherapy. Qual Life Res.

[45] Quintin C (2018). Determining clinically important differences in health-related quality of life in older patients with cancer undergoing chemotherapy or surgery. Qual Life Res.

[46] Kerezoudis P, et al. Defining the minimal clinically important difference for patients with vestibular Schwannoma: are all quality-of-life scores significant? Neurosurgery. 2018. 10.1093/neuros/nyy467. 10.1093/neuros/nyy467 30395303

[47] Soer R, Reneman MF, Speijer BL (2012). Clinimetric properties of the EuroQol-5D in patients with chronic low back pain. Spine J.

[48] Parker SL, Adogwa O, Mendenhall SK (2012). Determination of minimum clinically important difference (MCID) in pain, disability, and quality of life after revision fusion for symptomatic pseudoarthrosis. Spine J.

[49] Parker SL, Mendenhall SK, Shau DN (2012). Minimum clinically important difference in pain, disability, and quality of life after neural decompression and fusion for same-level recurrent lumbar stenosis: understanding clinical versus statistical significance. J Neurosurg Spine..

[50] Parker SL, Godil SS, Shau DN (2013). Assessment of the minimum clinically important difference in pain, disability, and quality of life after anterior cervical discectomy and fusion: clinical article. J Neurosurg Spine..

[51] Chuang LH, Garratt A (2013). Comparative responsiveness and minimal change of the knee quality of life 26-iten (KQoL-26) questionnaire. Qual Life Res.

[52] Díaz-Arribas MJ (2017). Minimal clinically important difference in quality of life for patients with low Back pain. Spine..

[53] Shi HY, Chang JK, Wong CY (2010). Responsiveness and minimal important differences after revision total hip arthroplasty. BMC Musculoskelet Disord.

[54] Solberg T, Johnsen LG (2013). Can we define success criteria for lumbar disc surgery? : Estimates for a substantial amount of improvement in core outcome measures. Acta Orthop.

[55] Carreon LY, Bratcher KR (2013). Differentiating minimum clinically important difference for primary and revision lumbar fusion surgeries. J Neurosurg Spine.

[56] Asher AL (2018). Defining the minimum clinically important difference for grade I degenerative lumbar spondylolisthesis: insights from the quality outcomes database. Neurosurg Focus.

[57] Kwakkenbos L (2013). A comparison of the measurement properties and estimation of minimal important differences of the EQ-5D and SF-6D utility measures in patients with systemic sclerosis. Clin Exp Rheumatol.

[58] Kohn CG, Sidovar MF (2014). Estimating a minimal clinically important difference for the EuroQol 5-dimension health status index in persons with multiple sclerosis. Health Qual Life Outcomes.

[59] Zhou F, Zhang Y (2015). Assessment of the minimum clinically important difference in neurological function and quality of life after surgery in cervical spondylotic myelopathy patients: a prospective cohort study. Eur Spine J.

[60] Fulk GD (2010). How much change in the stroke impact Scale-16 is important to people who have experienced a Storke?. Top Stroke Rehabil.

[61] Frans FA, Nieuwkerk PT (2014). Statistical or clinical improvement? Determining the minimally important difference for the vascular quality of life questionnaire in patients with critical limb ischemia. Eur J Vasc Endovasc Surg.

[62] Kim SK (2015). Estimation of minimally important differences in the EQ-5D and SF-6D indices and their utility in stroke. Health Qual Life Outcomes.

[63] Chen P, Lin KC (2016). Validity, responsiveness, and minimal clinically important difference of EQ-5D-5L in stroke patients undergoing rehabilitation. Qual Life Res.

[64] Yuksel S (2019). Minimum clinically important difference of the health-related quality of life scales in adult spinal deformity calculated by latent class analysis: is it appropriate to use the same values for surgical and nonsurgical patients?. Spine J.

[65] Le QA, Doctor JN, Zoellner LA (2013). Minimal clinically important differences for the EQ-5D and QWB-SA in post-traumatic stress disorder (PTSD): results from a doubly randomized preference trial (DRPT). Health Qual Life Outcomes.

[66] Thwin SS (2013). Assessment of the minimum clinically important difference in quality of life in schizophrenia measured by the quality of well-being scale and disease-specific measures. Psychiatry Res.

[67] Fallissard B (2015). Defining the minimal clinically important difference (MCID) of the Heinrichs-carpenter quality of life scale (QLS). Int J Methods Psychiatr Res.

[68] Stark RG, Reitmeir P, Leidl R, Konig HH (2010). Validity, reliability, and responsiveness of the EQ-5D in inflammatory bowel disease in Germany. Inflamm Bowel Dis.

[69] Basra MK, Salek MS (2015). Determining the minimal clinically important difference and responsiveness of the dermatology life quality index (DLQI): further data. Dermatology..

[70] Modi AC, Zeller MH (2011). The IWQOL-kids: establishing minimal clinically important difference scores and test-retest reliability. Int J Pediatr Obes.

[71] Newcombe PA, Sheffield JK, Chang AB (2011). Minimally important change in a parent-proxy quality-of-life questionnaire for pediatric chronic cough. Chest..

[72] Hilliard ME (2013). Identification of minimal clinically important difference scores of the PedsQL in children, adolescents, and young adults with type 1 and type 2 diabetes. Diabetes Care.

[73] Gravbrot N, Daniel FK, et al. The minimal clinically important difference of the anterior Skull Base nasal Inventory-12. Neurosurgery. 2018;83(2):277–80. 10.1093/neuros/nyx401. 10.1093/neuros/nyx401 28973679

[74] Hoehle LP (2018). Responsiveness and minimal clinically important difference for the EQ-5D in chronic rhinosinusitis. Rhinology..

[75] Akaberi A (2018). Determining the minimal clinically important difference for the PEmbQoL questionnaire, a measure of pulmonary embolism-specific quality of life. J Thromb Haemost.

[76] Alanne S, Roine RP (2015). Estimating the minimum important change in the 15D scores. Qual Life Res.

[77] Gatchel RJ, Mayer TG (2010). Testing minimal clinically important difference: consensus or conundrum?. Spine J.

[78] Kamper SJ, Maher CG, Mackay G (2009). Global rating of change scales: a review of strengths and weaknesses and considerations for design. J Man Manipulative Ther.

[79] Herrmann D (1995). Reporting current, past and changed health status: what we know about distortion. Med Care.

[80] Schwartz CE, Sprangers MAG (1999). Methodological approaches for assessing response shift in longitudinal health-related quality-of-life research. Soc Sci Med.

[81] Walters SJ, Brazier JE (2003). What is the relationship between the minimally important difference and health state utility values? The case of the SF-6D. Health Qual Life Outcomes.

[82] Cohen J (1977). Statistical power for the behavioral sciences.

[83] Hosmer DW, Lemeshow S (2000). Applied logistic regression.

[84] Norman GR, Sloan JA, Wyrwich KW (2003). Interpretation of changes in health-related quality of life: the remarkable universality of half a standard deviation. Med Care.

[85] Wang YC, Hart DL, Stratford PW (2011). Baseline dependency of minimal clinically important improvement. Phys Ther.

[86] Sloan JA (2005). Assessing the minimally clinically significant difference: scientific considerations, challenges and solutions. COPD..

